# On the impact of inequity on the attainment of health results in the African Region: a methodological exploration

**DOI:** 10.1186/s12939-026-02935-7

**Published:** 2026-07-02

**Authors:** Humphrey Karamagi, Sokona Sy, Joseph Mung’atu, Anabay Mamo, Bertha Kembabazi, Hillary Kipruto, Benson Droti

**Affiliations:** 1 World Health Organization Regional Office for Africa, Brazzaville, Republic of Congo; 2 Department of Statistics and Actuarial Science, Jomo Kenyatta University of Agriculture and Technology, Nairobi, Kenya

## Abstract

**Background:**

The core of Universal Health Coverage (UHC) is ensuring everyone can access essential health services without financial hardship, shifting focus from specific interventions to individual outcomes. Yet, in the WHO African region, there remains a critical gap in understanding the interrelations of the dimensions of health inequities with each other, and with health outcomes. This paper explores the interrelations among inequity drivers to inform their prioritization in each country of the region.

**Methods:**

This study analysed the association of the commonly used eight drivers of health inequity (independent variable): residence, ethnicity, occupation, gender, religion, education, socioeconomic status, and social capital, with differences in health results across countries (dependent variable). UHC indices served as the indicators for the dependent variable. Data came from the Demographic and Health Surveys with normalized indices for ethnicity and religion from prior studies. Missing values were imputed. Principal Component Analysis generated weighted composite indices, and inequity gaps were normalized to a 0–1 scale. Partial Least Squares regression examined relationships while addressing multicollinearity. Model validation used Root Mean Square Error of Prediction and explained variance, verified against known country-level inequity patterns.

**Results:**

Health inequity in the WHO African Region shows marked disparities, with an average composite gap of 0.49 (± 0.038) on a 0–1 scale. Angola (0.79), Kenya (0.68), and Chad (0.67) record the highest inequities, while Algeria and Equatorial Guinea (0.31) show greater equity. Ethnicity is the strongest driver (0.65), creating 30% more disparity than wealth (0.55) or social capital (0.56). Four latent variables emerged as drivers of inequity and explained up to 77% of the variance in UHC service coverage across countries. These are personal characteristics, geographic residence, social networks, and socioeconomic position.

**Conclusion:**

The major emerging drivers of inequity are not the same drivers of inequality. It is more targeted to focus on deciphering the four emerging latent variables as drivers of inequities to align with the need for person-centred health services required in the PHC approach for attaining UHC results - a shift away from the common programmatic focus of the eight drivers of inequality.

**Supplementary Information:**

The online version contains supplementary material available at https://doi.org/10.1186/s12939-026-02935-7.

## Introduction

The need to ensure everyone can access the essential services they need for their health and well-being without financial catastrophe is the central tenet of Universal Health Coverage (UHC) [1]. This emphasis on ‘everyone’ represents a fundamental pivot away from focusing on attaining outcomes for specific interventions, towards outcomes at the individual level. UHC can only be attained when everyone can access and use the services, they need for their health and wellbeing – irrespective of who they are. This is a conceptual shift that requires an increased focus on the individual and how well their health needs are addressed. At present, outcomes in health are measured against one or more of the ‘3E’s at the population level; effectiveness, efficiency, and equity [2, 3]. We instead need to focus more on how well individuals feel the services are responding to their needs (effectiveness), without unnecessary costs (efficiency), and in a manner fair to all (equity). Health equity is central to UHC attainment, as it ensures everyone can attain their full potential for health and wellbeing. Within the health sector in Africa, the pursuit of health equity has been a consistent theme since the Alma Ata conference where it was incorporated as part of the Primary Health Care approach [4]. Equity is specifically important as it is concerned with ensuring fairness in the distribution of access to services, reflected as the central promise of the 2030 Agenda to ‘Leave no one Behind’ [5].

**Fig. 1:**
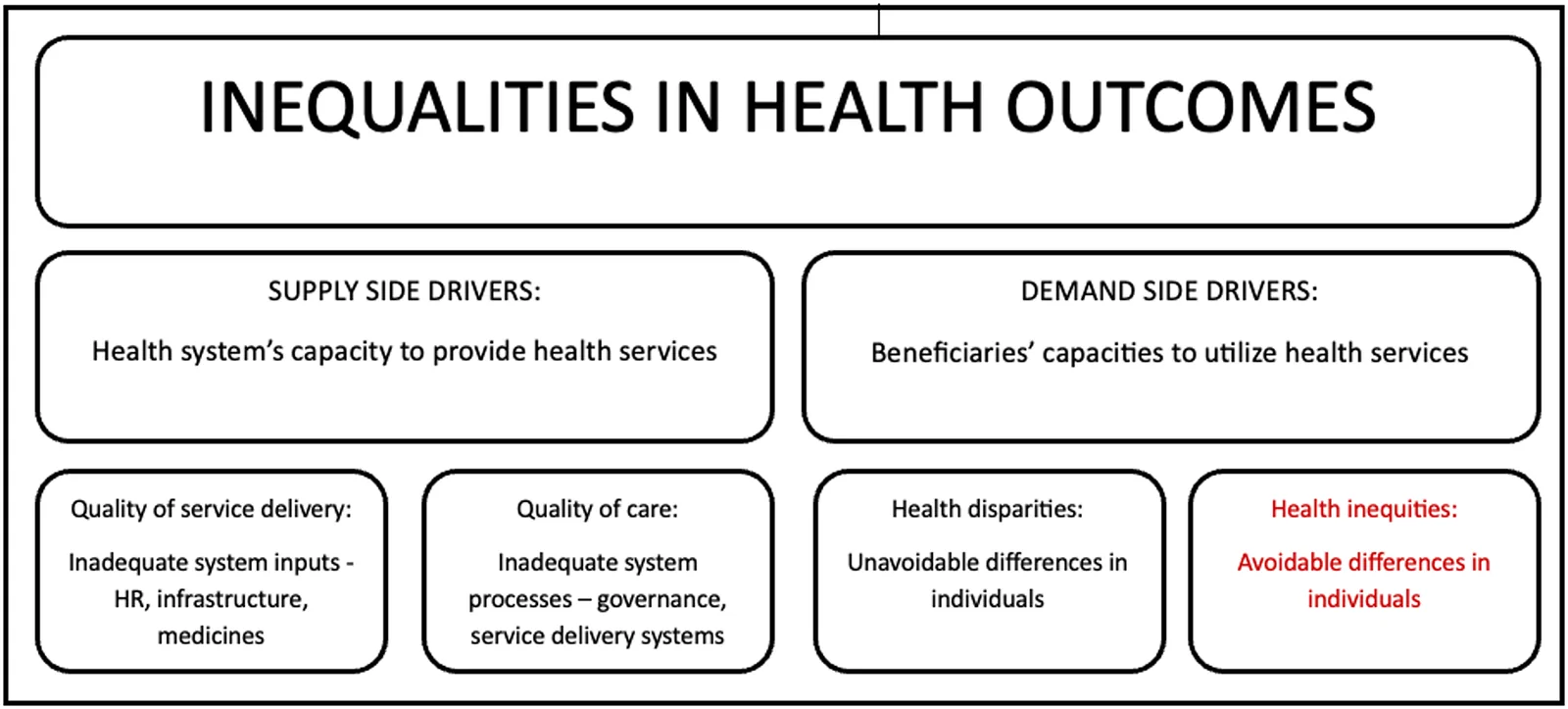
Conceptualizing drivers of health inequalities. Source: Authors own construction

In practice, the level of inequity is commonly based on the overall levels of inequality seen in health outcomes. Inequalities are a measure of the differences in health outcomes seen within and across given population groups. The level of health inequality drives the differences in health outcomes in defined populations. From the latest world health statistics, we see significant ranges of health outcomes and impact in African countries [6]. Healthy life expectancy ranges from 44.2 to 66.4 years; Maternal Mortality Rate (MMR) − 1,047 to 3 deaths per 100,000 live births; HIV incidence – 0.04 to 7.65 per 1,000 uninfected population; skilled birth attendance, 40–100%; and probability of dying from a noncommunicable disease between ages 30–70 years – 13.1–42.7% [6]. These variations in outcomes and impact are contributed to by both the differential access, and the differential capacities to utilize available services. This differential capacity to utilize services reflects the level of inequalities in a country. Health inequalities havemultiple drivers as shown below (see Fig. 1).

Health inequalities can be viewed as being driven by either supply, or demand side factors in each health sector. On the supply side, different levels of investment in the health system, and/or differences in how these investments are managed, lead to variations in access to essential services, and so different outcomes. On the other hand, the beneficiaries are also influenced on the demand side by either unavoidable, or avoidable drivers that influence their ability to utilize available services. Unavoidable drivers include issues like age, underlying genetics which hinder an individual’s availability to seek, access, or use health services. With health inequities, as are interested in the avoidable differences in individuals that influence health outcomes.

An analysis of health inequities therefore needs to first discount supply side gaps, plus the avoidable demand side influences in a given context. In practice, inequity is usually conflated with inequality, as it is perceived too complex to decipher from other drivers of inequality. From the literature, however, we identify 8 commonly used drivers of inequity: (1) place of residence, (2) race/ethnicity, (3) occupation, (4) gender/sex, (5) religion, (6) education, (7) socioeconomic status (wealth), and (8) social capital, commonly summarized with the acronym “PROGRESS” [7]. Looking at the literature, these eight ‘drivers of inequity’ are applied to different interventions and situations, often leading to competing prioritization for country decision-makers. Countries that attempt to address health inequities directly focus on specific drivers based on interests and funding, and not necessarily through a process to map the most important inequity drivers affecting them. These challenges – lack of specific focus on inequities, and selected action on specific inequity drivers – is contributing to the frustratingly poor progress in addressing inequities particularly in the African Region.

There remains a critical gap in understanding the interrelations of the dimensions of health inequalities with each other, and with health outcomes. This hinders the ability to design targeted and effective interventions to reduce health disparities. We postulate that, understanding the relative impacts of the different drivers of health inequity in each context will assist in implementing targeted interventions based on addressing specific drivers of inequity in each context. This will ensure health inequity actions are having the greatest possible impact on health outcomes. In this paper, we approach this by exploring the inter relations amongst the inequity drivers to generate information on their prioritization in each country of the WHO African region. The insights generated will guide policymakers in prioritizing and designing impactful interventions, ensuring progress towards equitable access to health services and the realization of UHC.

## Methodology

We identify consistent data on the different drivers of health inequalities that is representative of the countries in the WHO African Region, together with similarly consistent data on health outcomes. As the nature of the relationships across the drivers and with health outcomes is not clear, it is important to use a method capable of handling multicollinearity and high-dimensional data. By doing so, we aim to extract the complex relationships between drivers of inequity and quantify their relative contributions to health outcomes.

We consider the data on the different drivers of inequity to be independent variables, while that on health outcomes to be dependent variables. The dependent variable are the UHC variables; The dependent variables are the UHC Service Coverage Index, UHC service coverage sub-index on Service capacity & access; infectious diseases; Non-communicable diseases and Reproductive, maternal, newborn & child health. As such, independent variables are the latest data, by country, representing (1) place of residence, (2) race, analyzed as ethnicity, (3) occupation, (4) gender (sex), (5) religion, (6) education, (7) socioeconomic status analyzed as wealth, and (8) social capital. On the other hand, the dependent variables used in this study are the respective indices for UHC.

### Data sources and variables

For independent variables, the Demographic and Health Survey (DHS) database was used as the primary source of data, identifying indicators and data representing the state of the drivers of inequity, Appendix 3. Only 2 drivers of inequity, religion and ethnicity, did not have indicators within the DHS. For these, we used already analysed and normalized data on ethnic and religious fractionalization by country, produced using Fearon’s analysis [8] that compares the probability of heterogenicity on these two variables in each population [9–11]. The measure produces a relative normalized value for each country. To ensure comparability with the other drivers, the data were also summarized to produce relative normalized variations across countries. For the dependent variables, the World Health Statistics (2024) was used as the source for the Universal Health Coverage index by country [6].

For the 5 drivers of inequity that we relied on DHS as the data source, we extracted all indicators that presented data disaggregated by each stratifier. All the indicators with data disaggregation were included to avoid biases if we decided to use specific indicators. The number of indicators with this disaggregated data were as follows: place of residence (24), occupation (14), sex (18), education (19), wealth (27), and social capital (10) – see appendix 1 for the data and values by country.

### Data preparation

The DHS data was captured in an excel tables, showing indicator data by country. As the DHS data is not collected at the same time for all the countries, there were missing data points. Predictive imputation was applied to estimate missing data, ensuring the dataset’s completeness and reliability [12].

### Data analysis

The DHS data were processed in three stages to ensure comparability with the already normalized and relative data for the religion and ethnicity stratifiers.

Stage 1 – Deriving a single index value for each stratifier. The indices were derived using Principal Component Analysis (PCA), with factor loadings serving as weights among the independent variables [13] within each inequity dimension; e.g. Gender a value for male and one for female within each country.

Stage 2 – Deriving relative inequity gaps. This was done by determining the difference between the highest and lowest categories of the independent variable stratifiers. For education, this was the difference between the highest level attained and no education. Residence’s gap was between urban and rural indices was used. Wealth was analysed by comparing the highest and lowest quintiles. For gender, one sex served as the reference category, thus comparing males to females. In the cases of social capital and occupation, the indices were derived by linear combinations of the selected explanatory variables.

Stage 3 - Normalization of the inequity gaps: The relative inequity gaps were then normalized across the countries within a range of 0 to 1. Values close to 1 signified high inequities for the dimension and the converse meant less inequities for the specific country.

Correlation analysis was performed to establish the relationship between the relative inequity indices (independent variables) against health outcomes data (dependent variables) for each country: Partial Least Squares (PLS) regression was employed to model the relationships between multiple independent variables (inequities in social determinants) and multiple dependent variables (health and social outcomes). PLS is particularly effective in handling multicollinearity and high dimensional data [14]. Kernel PLS, a nonparametric method utilizing kernel functions to project input data into a higher dimensional feature space, was considered. This approach enables the capture of complex, nonlinear relationships between variables without imposing strong parametric assumptions [15].

The methodology takes the form of the measurement and structural model.

The measurement part of the model defines the relationships between the observed variables (independent variables) and the latent variables (components) as follows:

$$\eqalign{& {\rm{Latent}}\,{\rm{variabl}}{{\rm{e}}_i} \cr& = \sum\limits_{{\rm{j = 1}}}^{\rm{p}} {{\rm{Loadin}}{{\rm{g}}_{{\rm{ij}}}} \times {\rm{Independent}}\,{\rm{variabl}}{{\rm{e}}_{\rm{j}}}} \cr} $$

Where:

*Latent Variable*_*i*_ *-* is the i-th latent variable (component).

*Loading*_*ij*_ *-* is the loading of the j-th independent variable on the i-th latent variable.

*Independent Variable*_*j*_ *-* is the j-th independent variable (e.g., Education, Gender, etc.).

On the other hand, the structural part of the model defines the relationships between the latent variables and the dependent variables.

$$\eqalign{& {\rm{Dependent}}\,{\rm{variabl}}{{\rm{e}}_k} \cr& = \sum\limits_{{\rm{j = 1}}}^{\rm{m}} {{\rm{Coefficien}}{{\rm{t}}_{{\rm{ki}}}} \times {\rm{Latent}}\,{\rm{variabl}}{{\rm{e}}_{\rm{i}}}} \cr} $$

Where:

Dependent Variable_k_ - is the k-th dependent variable.

*Coefficient*_*ki*_ *-* is the coefficient of the i-th latent variable on the k-th dependent variable.

*Latent Variable*_*i −*_ is the i-th latent variable.

### Validation of methods

The emerging indices for the independent variables were broadly reviewed against expected patterns. For example, countries known to be mono-religious, such as Mali, or those with relatively homogenous socioeconomic profiles, such as Seychelles, were examined to assess the alignment of emerging inequity values with anticipated outcomes. The normalized relative indices were largely consistent with expectations. The validation of the Partial Least Squares (PLS) model was conducted using two critical measures: the Root Mean Squared Error of Prediction (RMSEP) and the variance explained by the components. RMSEP was utilized to quantify the predictive accuracy of the model, with lower RMSEP values indicating a higher degree of precision. The variance explained by the components was analysed to determine how effectively the predictors accounted for variations in the response variables. To ensure parsimony, the selection of the desired number of components focused on achieving a balance between explanatory power and simplicity. This was accomplished by identifying the point at which additional components contributed minimally to the model’s overall variance explained, thereby avoiding overfitting. These findings provided confidence that the emerging inequity values by country were representative of the actual situation in the WHO African Region. The results are presented by overall regional inequity value, by dimension of inequity, and by country. Additionally, the emergent relationships between the dimensions of inequity and health outcomes are explored.

### Ethics approval

Ethical approval was not required as this research does not involve human participants or the use of unpublished secondary data.

### Patient and public involvement

None.

### Data availability

The datasets used and/or analysed during the current study are available from the corresponding author on reasonable request.

## Results

### Aggregate inequity gaps per country (all the dimensions together)

**Fig. 2:**
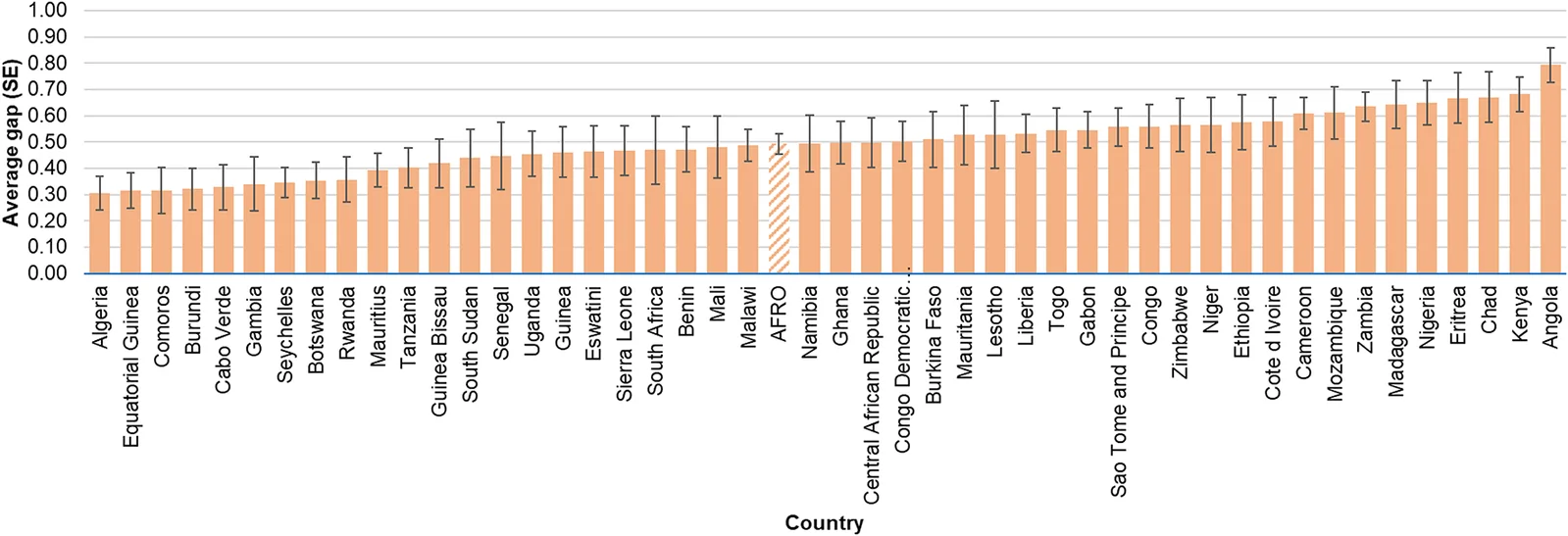
Average normalized gap from the eight inequity dimensions. Countries with the highest standard error (SE) in their inequity gaps include South Sudan (0.110), Lesotho (0.127), Senegal (0.128), South Africa (0.129), and Mali (0.117). These high SE values indicate significant variability across inequity dimensions, reflecting inconsistent impacts on health outcomes. In contrast, countries with the lowest standard error (SE) include Zambia (0.056), Seychelles (0.057), Cameroon (0.059), Malawi (0.061), and Algeria (0.064). The low SE values in these countries indicate more stable inequity profiles across dimensions, with relatively uniform impacts on health outcomes

In the WHO African Region (AFRO), the average inequity gap, from the eight [8] dimensions of inequity, stands at 0.49 (SE: ± 0.038), Fig. 2. The top five countries with the highest average inequity gaps are Angola (0.79, SE: ± 0.067), Kenya (0.68, SE: ± 0.066), Chad (0.67, SE: ± 0.095), Eritrea (0.67, SE: ± 0.095), and Nigeria (0.65, SE: ± 0.084). Conversely, the countries with the least average inequity gaps include Algeria (0.31, SE: ± 0.064), Equatorial Guinea (0.31, SE: ± 0.068), Comoros (0.31, SE: ± 0.088), Burundi (0.32, SE: ± 0.078), and Cabo Verde (0.33, SE: ± 0.087).

### Average inequity gaps by dimension

Ethnicity emerged as the most significant driver of inequities (0.65; 95% CI: 0.58–0.72), followed closely by social capital (0.56; 95% CI: 0.50–0.63) and wealth (0.55; 95% CI: 0.48–0.62). A middle tier of inequity drivers comprises religion (0.53; 95% CI: 0.46–0.61), occupation (0.49; 95% CI: 0.42–0.56), and residence (0.48; 95% CI: 0.40–0.57). Meanwhile, gender (0.37; 95% CI: 0.30–0.43) and education (0.34; 95% CI: 0.26–0.42) represent the relative lowest drivers of inequity (Appendix 2).

### Drivers of inequity by country

For the top five countries with the highest gaps, inequalities are predominantly driven by marked disparities in wealth and residence. For instance, Angola exhibits extreme inequity in gender and residence. Similarly, Chad and Eritrea face pronounced disparities in ethnicity, coupled with high levels of wealth inequity. Nigeria and Kenya demonstrate higher inequities across ethnicity and wealth dimensions, exacerbated by challenges in education and occupation, Table 1.

**Table 1:** The normalized inequity gaps with the ranks within each country

Country	Education	Gender	Ethnicity	Religion	Residence	Wealth	Social capital	Occupation
Algeria	0.2 (7)	0.24 (5)	0.34 (4)	0.01 (8)	0.2 (6)	**0.37** **(3)**	**0.58** **(1)**	**0.5** **(2)**
Angola	0.81 (4)	**1** **(1)**	0.79 (5)	0.63 (7)	**0.94** **(3)**	**0.97** **(2)**	0.43 (8)	0.78 (6)
Benin	0.12 (8)	0.52 (5)	**0.79** **(1)**	**0.55** **(3)**	0.35 (6)	0.55 (4)	**0.72** **(2)**	0.17 (7)
Botswana	0.11 (8)	0.26 (5)	0.41 (4)	**0.6** **(1)**	0.13 (7)	0.23 (6)	**0.58** **(2)**	**0.51** **(3)**
Burkina Faso	0.11 (7)	0.34 (6)	**0.74** **(2)**	0.58 (5)	0.66 (4)	**0.68** **(3)**	**0.88** **(1)**	0.08 (8)
Burundi	**0.42** **(3)**	0.26 (5)	0.3 (4)	**0.52** **(2)**	0 (8)	0.21 (6)	0.15 (7)	**0.71** **(1)**
Cabo Verde	0.22 (5)	0.03 (8)	0.42 (4)	0.08 (7)	0.13 (6)	**0.68** **(1)**	**0.57** **(2)**	**0.5** **(3)**
Cameroon	**0.66** **(3)**	0.34 (8)	**0.86** **(1)**	**0.73** **(2)**	0.61 (6)	0.64 (4)	0.62 (5)	0.4 (7)
Central African Republic	0.5 (6)	**0.57** **(3)**	**0.83** **(1)**	**0.79** **(2)**	0.02 (8)	0.23 (7)	0.55 (4)	0.5 (5)
Chad	**0.95** **(1)**	0.7 (5)	**0.86** **(2)**	0.64 (6)	0.82 (4)	0.33 (7)	**0.86** **(3)**	0.2 (8)
Comoros	0.27 (5)	0.39 (4)	0 (8)	0.01 (7)	0.15 (6)	**0.58** **(2)**	**0.66** **(1)**	**0.46** **(3)**
Congo	0.08 (8)	0.53 (5)	**0.87** **(1)**	**0.66** **(3)**	0.49 (6)	0.62 (4)	**0.75** **(2)**	0.48 (7)
Congo Democratic Republic	0.2 (8)	0.47 (5)	**0.87** **(1)**	**0.7** **(2)**	0.34 (7)	0.38 (6)	**0.54** **(3)**	0.52 (4)
Cote d Ivoire	0.17 (8)	0.48 (6)	**0.82** **(1)**	**0.76** **(3)**	0.75 (4)	0.66 (5)	**0.76** **(2)**	0.21 (7)
Equatorial Guinea	0.14 (6)	0.21 (5)	0.35 (4)	0.12 (8)	0.12 (7)	**0.49** **(3)**	**0.58** **(1)**	**0.51** **(2)**
Eritrea	0.48 (6)	0.24 (8)	0.65 (5)	0.43 (7)	**0.82** **(3)**	**0.98** **(2)**	0.73 (4)	**1** **(1)**
Eswatini	0.3 (6)	0.18 (7)	0.06 (8)	0.44 (5)	**0.79** **(2)**	**0.81** **(1)**	**0.64** **(3)**	0.5 (4)
Ethiopia	**0.87** **(2)**	0.21 (7)	**0.72** **(3)**	0.62 (5)	**0.92** **(1)**	0.39 (6)	0.13 (8)	0.72 (4)
Gabon	0.28 (8)	0.31 (7)	**0.77** **(1)**	0.67 (4)	0.38 (6)	0.57 (5)	**0.68** **(3)**	**0.7** **(2)**
Gambia	0.09 (8)	0.27 (4)	**0.79** **(1)**	0.1 (6)	**0.41** **(3)**	0.1 (7)	**0.77** **(2)**	0.21 (5)
Ghana	0.24 (7)	0.23 (8)	**0.67** **(3)**	**0.8** **(1)**	0.25 (6)	**0.71** **(2)**	0.51 (5)	0.57 (4)
Guinea	0.38 (4)	0.13 (8)	**0.74** **(3)**	0.26 (7)	**0.77** **(2)**	0.33 (5)	**0.82** **(1)**	0.26 (6)
Guinea Bissau	0.07 (8)	0.19 (6)	**0.81** **(1)**	**0.61** **(2)**	0.12 (7)	0.48 (5)	**0.57** **(3)**	0.5 (4)
Kenya	0.71 (4)	0.44 (7)	**0.86** **(2)**	**0.78** **(3)**	0.59 (6)	**0.95** **(1)**	0.43 (8)	0.7 (5)
Lesotho	0.29 (5)	0.01 (8)	0.26 (7)	0.72 (4)	**0.82** **(3)**	**1** **(1)**	0.28 (6)	**0.84** **(2)**
Liberia	0.49 (6)	**0.6** **(3)**	**0.91** **(1)**	0.49 (5)	0.46 (7)	0.18 (8)	0.49 (4)	**0.64** **(2)**
Madagascar	**1** **(1)**	0.42 (7)	**0.88** **(2)**	0.52 (6)	0.69 (4)	0.58 (5)	0.23 (8)	**0.82** **(3)**
Malawi	0.42 (6)	0.38 (7)	**0.67** **(2)**	**0.82** **(1)**	0.43 (4)	0.29 (8)	**0.45** **(3)**	0.43 (5)
Mali	0.19 (6)	**0.86** **(2)**	**0.69** **(3)**	0.18 (7)	0.56 (4)	0.37 (5)	**0.94** **(1)**	0.05 (8)
Mauritania	0.2 (7)	0.35 (6)	0.62 (4)	0.01 (8)	**0.82** **(2)**	**0.96** **(1)**	**0.68** **(3)**	0.57 (5)
Mauritius	0.22 (7)	0.35 (5)	0.46 (4)	**0.64** **(1)**	0.13 (8)	0.28 (6)	**0.57** **(2)**	**0.5** **(3)**
Mozambique	0.52 (7)	0.57 (6)	0.69 (4)	0.68 (5)	**0.96** **(1)**	**0.76** **(2)**	0 (8)	**0.7** **(3)**
Namibia	0.1 (7)	0 (8)	0.63 (4)	**0.66** **(3)**	0.45 (6)	**0.89** **(1)**	0.47 (5)	**0.75** **(2)**
Niger	0.62 (5)	0.47 (6)	0.65 (4)	0.2 (7)	**1** **(1)**	**0.66** **(3)**	**0.8** **(2)**	0.12 (8)
Nigeria	**0.83** **(2)**	0.64 (6)	**0.85** **(1)**	**0.74** **(3)**	0.62 (7)	0.7 (5)	0.72 (4)	0.09 (8)
Rwanda	0.05 (8)	0.14 (7)	0.32 (4)	**0.51** **(3)**	0.31 (5)	**0.56** **(2)**	0.19 (6)	**0.78** **(1)**
Sao Tome and Principe	0.3 (8)	0.4 (7)	**0.88** **(1)**	**0.78** **(2)**	0.41 (6)	**0.64** **(3)**	0.6 (4)	0.44 (5)
Senegal	0.05 (7)	0.45 (5)	**0.69** **(3)**	0.15 (6)	0.49 (4)	**0.75** **(2)**	**1** **(1)**	0 (8)
Seychelles	0.25 (5)	0.43 (4)	0.2 (7)	0.23 (6)	0.13 (8)	**0.47** **(3)**	**0.57** **(1)**	**0.5** **(2)**
Sierra Leone	0.34 (6)	**0.79** **(2)**	**0.82** **(1)**	0.54 (4)	0.36 (5)	0 (8)	**0.57** **(3)**	0.32 (7)
South Africa	0 (8)	0.08 (7)	**0.75** **(3)**	**0.86** **(2)**	0.2 (6)	0.69 (4)	0.29 (5)	**0.89** **(1)**
South Sudan	0.06 (8)	0.14 (6)	**0.85** **(1)**	**0.8** **(2)**	0.1 (7)	0.49 (5)	**0.57** **(3)**	0.5 (4)
Tanzania	0.05 (8)	0.24 (7)	**0.74** **(1)**	**0.63** **(2)**	0.42 (4)	0.38 (5)	**0.44** **(3)**	0.33 (6)
Togo	0.23 (7)	0.46 (6)	**0.71** **(3)**	0.66 (4)	**0.73** **(2)**	0.56 (5)	**0.82** **(1)**	0.19 (8)
Uganda	0.22 (7)	0.43 (4)	**0.93** **(1)**	**0.63** **(2)**	0.18 (8)	0.31 (6)	**0.52** **(3)**	0.42 (5)
Zambia	0.69 (4)	0.4 (8)	**0.78** **(2)**	**0.74** **(3)**	**0.84** **(1)**	0.55 (6)	0.45 (7)	0.63 (5)
Zimbabwe	0.61 (5)	0.15 (8)	0.39 (6)	**0.74** **(3)**	0.72 (4)	**0.87** **(1)**	0.2 (7)	**0.84** **(2)**

In the bottom five countries, while overall inequity levels are lower, key drivers persist. Algeria and Equatorial Guinea still experience significant disparities in social capital and occupation, while Burundi has imbalances related to ethnicity. Cabo Verde and Comoros, while maintaining lower inequity gaps, face challenges in wealth distribution and socioeconomic equity.

### Relationships between health outcomes and dimensions of inequity

To explain the relationship between the independent (dimension) and dependent (health outcomes – as measured by UHC service coverage) variables, the model derived eight latent components from the inequity dimensions. Each of these components are constructed from multiple actions within the dimensions of inequity. The 1st latent component contributes 27% of the contribution of inequity to health outcomes, while the 1st 5 components alone contribute 89% of the expected impact on health outcomes (see Table 2).

**Table 2:** The proportion of the response variance explained by the latent variables

Component	1 Comp	2 Comps	3 Comps	4 Comps	5 Comps	6 Comps	7 Comps	8 Comps
X	27.394	53.550	70.365	77.278	88.56	93.51	96.60	100.00
UHC Service Coverage Index,	36.359	36.530	37.550	37.813	38.03	38.77	38.80	38.80
UHC_ Service capacity & access	34.327	34.945	35.050	39.740	39.74	41.09	41.42	41.43
UHC_ infectious diseases	17.508	25.308	31.328	34.631	35.34	35.89	37.34	37.52
UHC_ Non-communicable diseases	3.363	3.935	6.068	6.162	17.41	19.75	22.17	22.22
UHC_ Reproductive, maternal, newborn & child health	17.149	21.511	22.133	24.344	24.45	24.71	24.71	24.72

### Loadings with the independent variables

Looking at the relation of these latent components and specific dimensions of inequity, we see the picture shown in Table 3. The model illustrates both positive, and negative relationships. Positive relationships suggest where an increase in the inequity gap correlates with an improvement in the health outcome, which could suggest that the better-off group (e.g., urban residents or those with higher education) disproportionately drives the health outcome. Conversely, negative relationships show widening the inequity gap (large gaps) correlates with poorer health outcomes, often reflecting the disadvantage faced by the less privileged group. The magnitude of these loadings demonstrates the strength of the predictor’s influence.

Residence has a dominant role (0.517) in the virtual Component 1, which impacts the first latent factor, reflecting its strong influence on health outcomes. Education (0.461 in Component 1) and Ethnicity (0.468 in Component 1) also show substantial contributions to the first component. Conversely, Social Capital exhibits variability, with a negative contribution (-0.500 in Component 2) highlighting its complex relationship with certain latent factors.

**Table 3:** The summary of factor loadings associated with the predictor variables

Predictor	Comp 1	Comp 2	Comp 3	Comp 4	Comp 5	Comp 6	Comp 7	Comp 8
Education	0.461	0.323	-0.243	-0.260	-0.599	-0.229	-0.358	0.547
Gender	0.427	-0.158	0	0	-0.289	-0.470	0.392	-0.702
Ethnicity	0.468	0.107	0.566	0	0.291	0.240	0.420	0
Religion	0.200	0.491	0.251	0.651	0.382	-0.501	-0.103	0
Residence	0.517	0.177	-0.768	-0.196	0.128	0.608	-0.287	-0.208
Wealth	0	0.276	-0.691	-0.539	0.473	-0.172	0.647	0
Social Capital	0.171	-0.500	0	-0.375	0.426	-0.212	-0.319	0
Occupation	-0.263	0.601	0	-0.354	-0.186	0	-0.148	-0.388

The interplay between predictors and components, as highlighted by these loadings, emphasizes the multidimensional nature of health inequalities. Strong loadings like those for Residence in Component 1 suggest an essential role in explaining health disparities, while fluctuating loadings for variables like Social Capital and Wealth highlight their complex, context-dependent contributions.

### Selection of the optimal latent variables

The RMSEP (Appendix 4) values for all response variables consistently support 4 components as the optimal number, which coincides with the “elbow” observed in the PCA scree plot. Beyond this point, the benefits of adding more components diminish, and in some cases, prediction errors increase. Therefore, components beyond 4 often lead to minimal improvements and risk overfitting.

**Fig. 3:**
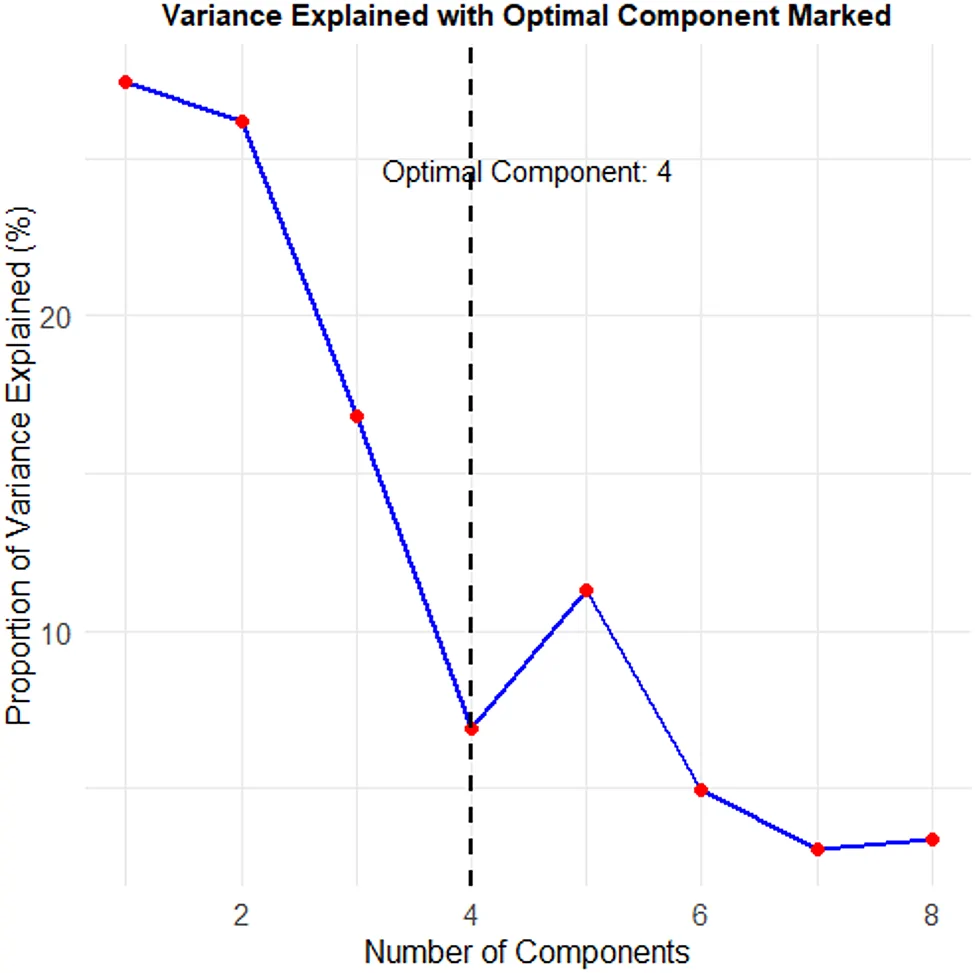
Proportion of the variance explained by the predictors

The variance explained by the predictors also arrived at the same conclusion as the gradient of the line changed after 4 components in Fig. 3.

The cumulative variance explained for 4 components is 77% - implying 77% of the variation in the predictor variables is captured by the model. This validation process ensures the PLS model generalizes well while identifying the key latent structures in the data. The explained variance is cumulatively as follows for the 8 components: 1 component: 27.39%, 2 components: 53.55%, 3 components: 65.36%, 4 components: 77.26%, 5 components: 88.92%, 6 components: 93.11%, 7 components: 96.93% and 8 components: 100.00%.

### The leading latent variables

**Fig. 4:**
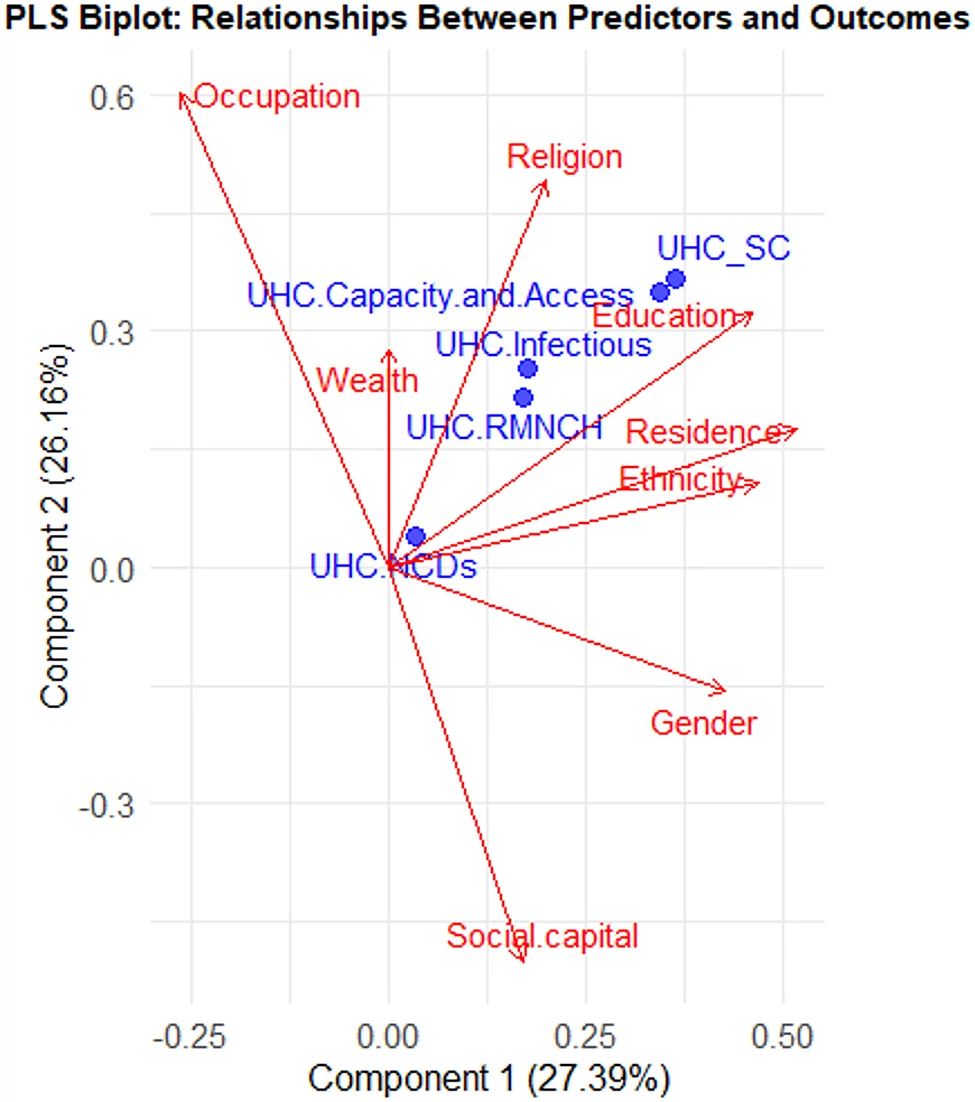
Ordination plot for the leading two components

To simplify complex relationships, we consider the first two components account which together account for 53.55% of the total variance (27.39% for Comp 1 and 26.16% for Comp 2), Fig. 4. This indicates that most healthcare disparities can be attributed to structural and socioeconomic inequity gaps. Beyond these components, additional components add marginal explanatory power, as seen in UHC Service Coverage Index variance increasing only slightly from 36.53% (2 components) to 38.80% (8 components). This reinforces the critical role of the first two components in capturing relationships between inequity gaps and health outcomes.

## Discussion

The outcomes from this study highlights the complexity around health equity actions in different countries and programs, if it is to facilitate progress toward universal health coverage. Most commonly, we have used health inequality as a proxy measure for health inequity, as it is generally considered too complex to monitor health inequities. However, we can decipher the actual contribution of inequities as contributors to inequality. From the inequity lens, the study shows the most influential and context-specific drivers of disparities are more related to ethnicity, social capital, and wealth, which are not fully aligned with to the drivers we commonly focus on when we use the inequality proxy – particularly gender and education.

Ethnicity is shown to be the strongest and most consistent contributor to health inequity. This is not surprising in a continent home to hundreds of ethnic groups, where ethnic identity is both a source of belonging and a potential axis of exclusion. Ethnic identity in Africa is not inherently divisive, but can become salient when it is mobilized for access to resources [16]. To this end, historical patterns of ethnic favoritism perpetuated through uneven development continue to shape how services are allocated and who benefits from them [17, 18]. Several studies assessing health service indicators disaggregated by ethnicity found that majority groups were consistently more likely to access services [19]. These findings underscore the need for targeted and culturally responsive approaches to ensure that populations of different ethnicities are not left behind.

Social capital emerged as the second strongest, but context-dependent contributor to health inequities. In many African cultures, strong social networks are the norm and serve as a fulcrum for community support structures that can facilitate access to health services by enabling trust, information sharing, and informal assistance. Collective values and mutual responsibility remain common denominators in African culture. However, the role of social capital is not uniformly positive. In Ethiopia, for example, strong community ties were shown to reinforce social pressure for women to give birth at home rather than in health facilities, illustrating how collective norms can sometimes conflict with public health goals [20]. These findings remind us that while social capital can be a positive force, it needs to be aligned with public health goals.

Wealth emerged as the third strongest driver of health inequities, but is not context dependent. Numerous studies confirm that wealthier individuals consistently have higher service coverage [19]. However, in some services, such as immunization, ITNs, and ART, which are typically provided free of charge, coverage was often higher among poorer groups, suggesting that equality in financial access significantly reduces health inequities in utilization [19]. An analysis of 28 RMNCH indicators found that 57% showed narrowing wealth-related gaps, largely due to health insurance schemes, fee waivers, and service exemptions. Still, 42% of indicators showed persistent or widening disparities, despite national equity strategies [19]. This indicates that financial reforms alone are not sufficient; barriers such as distance to facilities, quality of care, and fear of side effects or follow-up costs continue to limit access for the poorest populations.

The data on specific countries shows significant variations in the drivers of health inequalities, impacting the overall health inequity drivers. In Angola for instance, gender emerged as a significant inequality driver, despite significant levels of female representation in national leadership. This highlights the fallacy with a focus on inequality to understand inequities in health. Evidence supports this view that women’s presence in formal institutions - a means to improve gender inequality - does not translate into improved access, outcomes or empowerment – means to improve inequity [21]. Similarly, when we look at social capital as a source of inequalities, we see countries such as Burundi, Mozambique, Rwanda, and São Tomé and Príncipe do not rate it highly, while it was found to be a major driver of inequities. To improve social capital as a driver of inequity, it is important to focus more on community networks and collective values often associated with *Africanness* in these settings.

The analysis of the links between drivers of inequity and health outcomes led to four emergency latent variables constructed from the different drivers of inequity, that work together to impact on health results. These four latent variables represent a condensation of the impact of inequities within the inequality space, on health results. The first latent variable reflects personal character, and is driven by education, and wealth predictors. The second latent variable reflects the role of where you live and emphasizes the impact of residence. The third latent variable encapsulates the influence of who you live with and is driven by ethnicity and religion. Finally, the fourth latent variable reflects your station in life, and is driven by wealth, and religion predictors.

This emergent latent variable description has been proposed in other literature. For example., Dahlgren and Whitehead’s ‘Rainbow Model’, portrays how individual health is shaped by layers of influence, ranging from personal lifestyle factors to social and community networks, living and working conditions, and the broader socio-economic, cultural, and environmental context [22]. These layered determinants align closely with the latent variables identified in this study, reinforcing that health inequities are not random but systematically produced through structural and social arrangements. Furthermore, the latent variables also mirror the Diderichsen model of the mechanisms of health inequality, which explains how social contexts produce stratification, placing individuals in different social positions [23]. These positions determine differential exposure to health risks, differential vulnerability based on available resources and resilience, and differential consequences of ill health, where disadvantaged groups face greater economic and social harm. Finally, the latent variables are consistent with the WHO Commission on Social Determinants of Health [24], which emphasized that health inequities arise from the “conditions in which people are born, grow, live, work and age,” shaped by the unequal distribution of power, resources, and opportunity.

The latent variables relating to personal character, where you live the influence of who you live with, and your station in life present a more condensed perspective on health inequity within the African Region. By approaching health inequity from this lens, countries can tailor their approaches to addressing health inequity in a comprehensive manner, instead of focusing on specific standalone drivers of inequality. Each country can focus on the specific latent variable that it is lagging in, based on its score with the drivers of inequity.

### Study limitations

The study presents an innovative and comprehensive approach to using existing data on health inequalities, to better understand underlying inequities in health within the African Region. It would be useful to explore the emerging results and recommendations particularly from the potential impact on health outcomes.

Use of secondary data means interpretation is limited to the data design. Data on the stratification of health outcomes as per the different levels of inequalities within was not available for instance but would have greatly enhanced the findings of quality.

## Conclusion

Health inequity is recognized as an important attribute to address if we are to move towards the health results that countries in the African region aspire for. The analysis of inequities has in the past been hampered by the lack of specific data, relying more on inequality information as a proxy. In this study, we have shown that it is possible to tease out the effects of inequities on a country-by-country basis and derive specific priorities for focus. The major emerging drivers of inequity – ethnicity, social capital and wealth – are not necessarily the same drivers of inequality – education, gender, and residence. The emergent four latent variables driving inequities’ impacts on health results – that is, one’s character, where you live the influence of who you live with, and your station in life - align with the need for person-centred health services, as these latent variables talk to the individual better than the programmatic focus of the eight drivers of inequality.

Moving forward, it is important to test the implications of addressing the four latent variables based on the country-specific priority drivers, to quantify their respective impacts on health results.

## Supplementary Information

Below is the link to the electronic supplementary material.

Supplementary Material 1

Supplementary Material 2

Supplementary Material 3

Supplementary Material 4

## Data Availability

The data analysed in this study were obtained from the Demographic and Health Surveys (DHS) database and the World Health Organization’s World Health Statistics database. DHS datasets are publicly available upon registration. World Health Statistics data are publicly accessible through the WHO Global Health Observatory. The specific datasets used are described in the Methods section. No new primary data were generated for this study.
