# Supplementary material for: On the impact of inequity on the attainment of health results in the African Region: a methodological exploration
Source: Int J Equity Health. 2026 Jul 2;25:221. doi: 10.1186/s12939-026-02935-7 (PMC13602494; doi:10.1186/s12939-026-02935-7)
Supplement: Supplementary file 1 [file 12939_2026_2935_MOESM1_ESM.docx]

**Appendix 1: Variable Mapping Across Domains of Inequality**

1. **Education index variables**

The variables drawn from DHS and stratified by educational attainment (no education, primary, secondary, and higher) are: Unmet need for family planning, Demand for family planning satisfied by modern methods, Maternal mortality ratio, Place of delivery in a health facility, Received all 8 basic vaccinations, Received all 8 basic vaccinations in first year, Children underweight, Comprehensive correct knowledge about AIDS (Women), Comprehensive correct knowledge about AIDS (Men), HIV prevalence among men, HIV prevalence among general population, Women who own a mobile phone, Men who own a mobile phone, Ever experienced physical violence since age 18, Physical or sexual violence committed by husband/partner, Committed physical violence against their husband/partner ever, Women circumcised, Women who worked in the last 12 months and are currently employed, Men who worked in the last 12 months and are currently employed.

2. **Gender index variables**

Based on gender classification into Male and Female, the following variables were used to highlight differences across demographic, health, and behavioural indicators: Life expectancy at birth (years), Healthy life expectancy at birth (years), TB incidence, Adult mortality rate, Literacy status, Comprehensive correct knowledge about AIDS, Did no work in the last 12 months, Employed in the last 12 months, Ever used the internet, Have heard of HIV or AIDS, Higher-risk sex in the last year, HIV prevalence, Knowledge of where to get an HIV test, Knowledge of any method of contraception, No health insurance, Own a mobile phone, Use of any type of tobacco, and HIV prevalence differential. These indicators—derived from DHS datasets—enable gender-disaggregated analysis to inform targeted strategies in public health programming and monitoring.

3. **Wealth index variables**

Based on the wealth quintiles - Lowest, Middle, and Highest - disparities were examined across multiple domains using variables drawn from DHS and complementary system metrics. In the area of maternal and child health, the variables included women who did no work in the last 12 months, those categorized as obese according to BMI ≥ 30.0, access to antenatal care and number of visits (≥8), lack of antenatal care and postnatal checkups, and receipt of age-appropriate vaccinations (24–35 months). For disease prevention and vector control, indicators focused on household access to insecticide-treated mosquito nets (ITNs), their mean number per household, and usage the previous night. Health awareness and HIV knowledge were gauged through men's recognition of HIV or AIDS, understanding of HIV prevention methods (based on a composite of prompted components), and comprehensive correct knowledge about AIDS. Household infrastructure and basic services were reflected in water access (basic vs. limited), electricity coverage, and use of clean fuel for cooking by both households and individuals. Demographic and systemic indicators encompassed total population, income group, and land area. System-level metrics related to Universal Health Coverage (UHC) were addressed using service coverage for general health and NCDs, as well as system capacity. Finally, health status was measured through life expectancy at birth (LEB), healthy life expectancy at birth (HLEB), maternal mortality ratio (MMR), and skilled birth attendance (SBA).

4. **Residence index variables**

Based on residential classification - Rural or Urban - the following variables were used to highlight disparities in health outcomes, service utilization, literacy, labour participation, and household conditions, drawing primarily from DHS sources. In the domain of reproductive, maternal, and child health, differences were captured through unmet need for family planning, demand for family planning satisfied by modern methods, under-five mortality rate, maternal mortality ratio, place of delivery in a health facility, and receipt of all eight basic vaccinations - including those administered within the first year. Nutritional status and vector control were explored through indicators on children who were underweight and children under five who slept under an insecticide-treated net. HIV-related knowledge and prevalence metrics included comprehensive correct knowledge about AIDS among women and men, HIV prevalence among men and in the general population, and HIV prevalence differential. Gender-based violence indicators included experiences of physical violence since age 16, physical or sexual violence committed by a husband or partner, and instances where women reported committing physical violence against their partner. Educational and economic dimensions were reflected through literacy levels among women and men, current employment status of those who worked in the last 12 months (disaggregated by gender), and household-level infrastructure metrics such as households with electricity, population with electricity, and the mean number of household members. Together, these variables support comparative analysis of rural–urban inequities across health and social systems, and offer actionable insights for targeted interventions and planning.

5. **Social capital index variables**

For the domain of social capital, the variables assessed joint decision-making and asset ownership across gendered dimensions of household agency. Specifically, the indicators included: ownership of a house jointly by women and by men, ownership of land jointly by women and by men, joint decision-making between wife and husband regarding the woman’s own health care (as reported by both women and men), joint decision-making about major household purchases (reported separately by women and men), and joint decision-making about visits to her family or relatives (reported by women). These measures reflect the extent of shared agency and resource control within households, offering insights into gender equity and collaborative participation in domestic life - an essential layer of analysis for understanding empowerment pathways within population and health surveys.

6. **Ethnicity** **index variables**

In the domain of ethnicity, the analysis adopts two structural diversity indicators ethnic fractionalization and linguistic fractionalization. These measures quantify the probability that two randomly selected individuals from a population belong to different ethnic or linguistic groups, respectively.

7. **Religion index variables**

The analysis considered indicators that capture structural and demographic religious composition. These included religious fractionalization, which measures the probability that two randomly selected individuals belong to different religious groups, and disaggregated population shares: percent Muslim, percent Christian, and percent belonging to other religions.

8. **Occupation index variables**

The index was modelled using three core labour status indicators: Employed for kind, which captures individuals who worked and received remuneration in cash or kind; Employed, reflecting the broader definition of those currently engaged in any form of work; and Unemployed, representing individuals without current employment who may be actively seeking work or available for employment.
