# Supplementary material for: On the impact of inequity on the attainment of health results in the African Region: a methodological exploration
Source: Int J Equity Health. 2026 Jul 2;25:221. doi: 10.1186/s12939-026-02935-7 (PMC13602494; doi:10.1186/s12939-026-02935-7)
Supplement: Supplementary file 3 [file 12939_2026_2935_MOESM3_ESM.docx]

**Appendix 3: Latest DHS Surveys by Country**

| **Country** | **Latest DHS Survey** | **Year(s)** |
| --- | --- | --- |
| Algeria | No DHS (uses MICS/other surveys) | – |
| Angola | DHS (IIMS) | 2023-2024 |
| Benin | DHS | 2017-2018 |
| Botswana | DHS (and data from Central Statistics Office (Botswana)) | 1988 |
| Burkina Faso | DHS | 2021 |
| Burundi | DHS | 2016-2017 |
| Cabo Verde | DHS | 2005 |
| Cameroon | DHS | 2022 |
| Central African Republic | DHS | 2010 |
| Chad | DHS | 2014-2015 |
| Comoros | DHS | 2012 |
| Congo (Republic) | DHS | 2011-2012 |
| Congo (DRC) | DHS (EDS‑RDC II) | 2023-2024 |
| Côte d’Ivoire | DHS | 2021 |
| Equatorial Guinea | DHS | 2011 |
| Eritrea | DHS | 2002 |
| Eswatini | DHS | 2006-2007 |
| Ethiopia | DHS | 2021-2022 |
| Gabon | DHS | 2019-2021 |
| Gambia | DHS | 2019-2020 |
| Ghana | DHS | 2022 |
| Guinea | DHS | 2021 |
| Guinea‑Bissau | No DHS (MICS is used) | – |
| Kenya | DHS | 2022 |
| Lesotho | DHS | 2023-2024 |
| Liberia | DHS | 2022 |
| Madagascar | DHS | 2021 |
| Malawi | DHS | 2024 |
| Mali | DHS (EDSM‑VII) | 2023-2024 |
| Mauritania | DHS | 2019-2021 |
| Mauritius | No DHS | – |
| Mozambique | DHS | 2022 |
| Namibia | DHS | 2013 |
| Niger | DHS | 2021 |
| Nigeria | DHS | 2023–2024 |
| Rwanda | DHS | 2023 |
| São Tomé and Príncipe | DHS | 2014 |
| Senegal | DHS | 2023 |
| Seychelles | No DHS | – |
| Sierra Leone | DHS | 2019 |
| South Africa | DHS | 2016 |
| South Sudan | No DHS (uses MICS, other surveys) | – |
| Tanzania | DHS | 2022 |
| Togo | DHS | 2017 |
| Uganda | DHS | 2016 |
| Zambia | DHS | 2024 |
| Zimbabwe | DHS | 2023–2024 |
