# Supplementary material for: On the impact of inequity on the attainment of health results in the African Region: a methodological exploration
Source: Int J Equity Health. 2026 Jul 2;25:221. doi: 10.1186/s12939-026-02935-7 (PMC13602494; doi:10.1186/s12939-026-02935-7)
Supplement: Supplementary file 4 [file 12939_2026_2935_MOESM4_ESM.docx]

**Appendix 4: RMSEP (Root Mean Squared Error of Prediction) for the various models**

| Number of Components | UHC Service Coverage | UHC Capacity and Access | UHC Infectious diseases | UHC NCDs | UHC RMNCH |
| --- | --- | --- | --- | --- | --- |
| Intercept | 11.19 | 19.4 | 13.91 | 6 | 13.37 |
| 1 Component | 9.527 | 16.72 | 13.33 | 6.032 | 12.72 |
| 2 Components | 9.588 | 16.82 | **12.91** | 6.177 | 12.77 |
| 3 Components | 9.863 | 17.06 | 13.66 | **6.183** | 13.2 |
| 4 Components | **9.863** | **16.62** | 13.7 | 6.519 | **13.38** |
| 5 Components | 10.14 | 17.27 | 13.43 | 6.417 | 13.49 |
| 6 Components | 10.39 | 18.34 | 13.26 | 6.259 | 13.56 |
| 7 Components | 10.39 | 18.35 | 13.08 | 6.261 | 13.92 |
| 8 Components | 10.45 | 18.46 | 13.11 | 6.408 | 14.15 |

Notes

1. UHC SC RMSEP decreases significantly with the addition of the first component (from 11.19 to 9.527) and stabilizes around 4 components.
2. Service access: RMSEP drops significantly from 19.4 (Intercept) to 16.62 (4 components).
3. Infectious: RMSEP decreases steadily, with significant improvement at 2 components (12.91).
4. NCD RMSEP decreases slightly between 1-3 components but jumps at 4 components (6.519), indicating complexity or instability in prediction.
5. RMNCH: RMSEP decreases significantly with the first component (12.72) and stabilizes after 4 components (13.38).
