# Supplementary material for: On the impact of inequity on the attainment of health results in the African Region: a methodological exploration
Source: Int J Equity Health. 2026 Jul 2;25:221. doi: 10.1186/s12939-026-02935-7 (PMC13602494; doi:10.1186/s12939-026-02935-7)
Supplement: Supplementary file 5 [file 12939_2026_2935_MOESM2_ESM.docx]

**Appendix 2: Average inequity gaps by dimension**
